# The IGF-1 receptor inhibitor picropodophyllin potentiates the anti-myeloma activity of a BH3-mimetic

**DOI:** 10.18632/oncotarget.1933

**Published:** 2014-04-30

**Authors:** Liesbeth Bieghs, Susanne Lub, Karel Fostier, Ken Maes, Els Van Valckenborgh, Eline Menu, Hans E. Johnsen, Michael T. Overgaard, Olle Larsson, Magnus Axelson, Mette Nyegaard, Rik Schots, Helena Jernberg-Wiklund, Karin Vanderkerken, Elke De Bruyne

**Affiliations:** ^1^ Department of Hematology and Immunology-Myeloma Center Brussel, Vrije Universiteit Brussel, Brussels, Belgium; ^2^ Department of Haematology, Aalborg Hospital, Aalborg University, Denmark; ^3^ Department of Biomedicine, Aarhus University, Aarhus, Denmark; ^4^ Department of Chemistry and Biotechnology, Aalborg University, Denmark; ^5^ Department of Oncology and Pathology, Cancer Center Karolinska, Karolinska Institute, Stockholm, Sweden; ^6^ Department of Clinical Chemistry, Karolinska Hospital, Stockholm, Sweden; ^7^ Department of Immunology, Genetics and Pathology, Rudbeck Laboratory, Uppsala, Sweden

**Keywords:** Multiple myeloma, IGF-1 receptor inhibitor, BH3-mimetic, preclinical study, mouse model

## Abstract

The ABT-analogous 737, 263 and 199 are BH3 mimetics showing potent anti-myeloma (MM) activity, but only on defined molecular subgroups of MM patients presenting a Bcl-2^high^/Mcl-1^low^ profile. IGF-1 is a major survival factor in MM regulating the expression of Bcl-2 proteins and might therefore be a resistance factor to these ABT-analogous. We first show that IGF-1 protected human MM cell lines (HMCLs) against ABT-737. Concurrently, the IGF-1 receptor inhibitor picropodophyllin (PPP) synergistically sensitized HMCL, primary human MM and murine 5T33MM cells to ABT-737 and ABT-199 by further decreasing cell viability and enhancing apoptosis. Knockdown of Bcl-2 by shRNA protected MM cells to ABT-737, while Mcl-1 shRNA sensitized the cells. PPP overcame the Bcl-2 dependency of ABT-737, but failed to completely overcome the protective effect of Mcl-1. In vivo, co-treatment of 5T33MM bearing mice significantly decreased tumor burden and prolonged overall survival both in a prophylactic and therapeutic setting. Interestingly, proteasome inhibitor resistant CD138− 5T33MM cells were more sensitive to ABT-737, whereas PPP alone targeted the CD138+ cells more effectively. After co-treatment, both subpopulations were targeted equally. Together, the combination of an IGF-1R inhibitor and an ABT-analogue displays synergistic anti-myeloma activity providing the rational for further (pre)clinical testing.

## INTRODUCTION

Multiple myeloma (MM) is a hematological cancer characterized by the accumulation of malignant plasma cells in the bone marrow (BM). Direct and indirect interactions between the MM cells and the BM-microenvironment are important for MM pathogenesis, driving MM cell survival, growth, migration, drug resistance and immune escape. Despite the introduction of drugs targeting both the MM cells and these interactions in the clinic (e.g. bortezomib and lenalidomide), drug resistance inevitably develops, resulting in relapse of virtually all patients [1].

Drug resistance in cancer and MM has partly been assigned to elevated expression of the anti-apoptotic subfamily of Bcl-2 proteins. Three subfamilies can be defined, namely the anti-apoptotic (e.g. Bcl-2, Bcl-Xl, Mcl-1), pro-apoptotic Bax/Bak-like and pro-apoptotic BH3-only (e.g. Bim, Bad, Noxa) protein subfamily [2, 3]. The balance of these Bcl-2 proteins determines if a cell will undergo apoptosis. In light of the importance of the Bcl-2 family in drug resistance, BH3-mimetics have been designed. BH3-mimetics bind with high affinity to anti-apoptotic proteins, thereby preventing sequestration of the pro-apoptotic proteins and shifting the balance towards apoptosis. ABT-737 and its orally bioavailable analogue ABT-263 are potent, selective small-molecule inhibitors of Bcl-2 and Bcl-Xl, but not Mcl-1, showing potent clinical activity towards several hematological malignancies [3-5]. However, thrombocytopenia caused by Bcl-Xl inhibition proved to be the dose-limiting toxicity, thus limiting the efficacy of ABT-737 and -263 [6, 7]. To solve this, a new orally available high-affinity Bcl-2 selective BH3 mimetic, namely ABT-199, was developed only very recently and was reported to be at least as effective as ABT-737 and -263 without eliciting thrombocytopenia [8, 9]. In MM, however, both ABT-737 and -199 are only highly effective against one molecular subgroup with a neutral prognostic value, namely the CCND1 subgroup, of which the MM cells present a Bcl-2^high^/Mcl-1^low^ profile and depend on Bcl-2 for survival [10-12]. Since this CCND1 subgroup represents only a small fraction of all MM cases, the ABT-analogous thus hold little promise as single agents in most MM cases. We and others showed that IGF-1 is one of the two major growth and survival factors in MM, regulating the expression of Bcl-2 family members [13-17]. Moreover, the IGF-1 receptor (IGF-1R) is highly expressed on MM cells correlating with poor prognosis and is therefore an attractive target in MM [17]. Previously, we demonstrated that picropodophyllin (PPP) suppresses the IGF-1R tyrosine kinase activity and has potent anti-myeloma activity both in human MM cells and the murine 5TMM models [18, 19]. We hypothesized that IGF-1 might antagonize the effect of ABT-analogous in MM and that IGF-1R targeting therefore might overcome this resistance. As a proof of concept, we decided to use the most studied and characterized BH3 mimetic and ABT-analogue, namely ABT-737. We first investigated the protective effect of growth factors in general and more specifically IGF-1 against ABT-737 mediated MM cell death. Next, we evaluated the potential synergistic anti-MM effect of ABT-737 and PPP using human MM cell lines (HMCLs), primary human samples and the murine 5T33MM model.

## RESULTS

### IGF-1 protects against ABT-737 induced MM cell death

**Figure 1:**
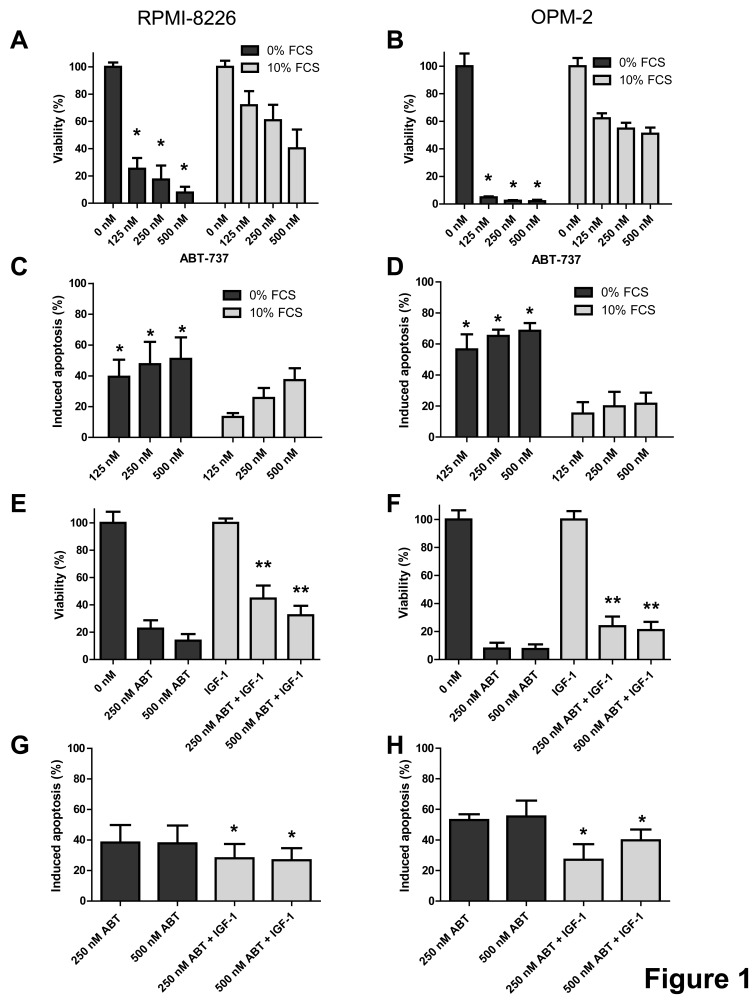
IGF-1 protects myeloma cells against ABT-737 induced cell death A-D: Serum protects MM cells to ABT-737. RPMI-8226 (A, C) and OPM-2 (B, D) cells were cultured in RPMI-1640 medium supplemented with or without 10% FCS and treated with different concentrations of ABT-737 (125, 250 and 500 nM). (A, B): The effect on cell viability was evaluated after 48h by using a CellTiter-Glo Assay. Results are expressed as the relative viability compared to untreated cells assayed in serum free medium or complete growth medium. (C, D): The effect on apoptosis was evaluated after 48h by an AnnexinV-FITC/7′AAD staining followed by FACS analysis. Results are expressed as the percentage induced apoptosis compared to control cells cultured in serum free medium or complete growth medium. Columns and error bars indicate mean ± SD of at least 4 independent experiments. * indicate p-values of ≤0.05 comparing treatment in serum free medium to treatment in complete growth medium. E-H: IGF-1 protects MM cells to ABT-737. RPMI-8226 (E, G) and OPM-2 (F, H) cells were serum starved overnight and pre-stimulated or not with IGF-1 (200 ng/ml) for 3h before ABT-737 (250 and 500 nM) was added. (E, F): Effect on cell viability after 48h was evaluated by using a CellTiter-Glo Assay. Results are expressed as the relative number of viable cells compared to control cells or IGF-1 condition. (G, H): Effect on apoptosis after 48h was determined by an AnnexinV-FITC/7′AAD staining followed by FACS analysis. Results are expressed as the percentage induced apoptosis compared to control cells or IGF-1 condition. Columns and error bars are the mean ± SD of at least 4 individual experiments. * and ** indicate p-values of respectively ≤0.05 and ≤0.01 comparing ABT-737 treatment with or without IGF-1.

In an initial set of experiments, we compared the effect of ABT-737 on MM cell viability and apoptosis in complete growth versus serum-free medium using two HMCLs that are described to show intermediate to low sensitivity to the ABT-analogous, namely RPMI-8226 and OPM-2 cells. In both cell lines, an increased response to ABT-737 was observed when assayed under serum-free conditions (Figure 1 A-D). At 48 hours, we observed a simultaneous decrease in viability and increase in apoptosis in the serum-free conditions, while observing only minimal cytotoxicity of ABT-737 in complete growth medium. The absolute number of apoptotic cells in the control cells when treated in complete growth medium was consistently below 20% for both cell lines. In addition, the difference in % apoptosis of myeloma cell lines in absence of treatment with or without serum was only minimal (less than 5 to 10%). Since IGF-1 is one of the two major growth factors in MM, we then tested the possibility that IGF-1 might protect MM cells against ABT-737 induced cell death. Cells were serum starved overnight and pre-stimulated with IGF-1 (200 ng/ml) before ABT-737 was added. Addition of IGF-1 partially protected against ABT-737 as evidenced by a significant increase in the number of viable cells and decrease in apoptosis compared to cells treated with ABT-737 alone for 48 hours (Figure 1 E-H). Together, these data show that IGF-1 exerts a partial protective effect against ABT-737 induced MM cell death.

### The IGF-1R inhibitor PPP potentiates the anti-myeloma effect of ABT-737

**Figure 2:**
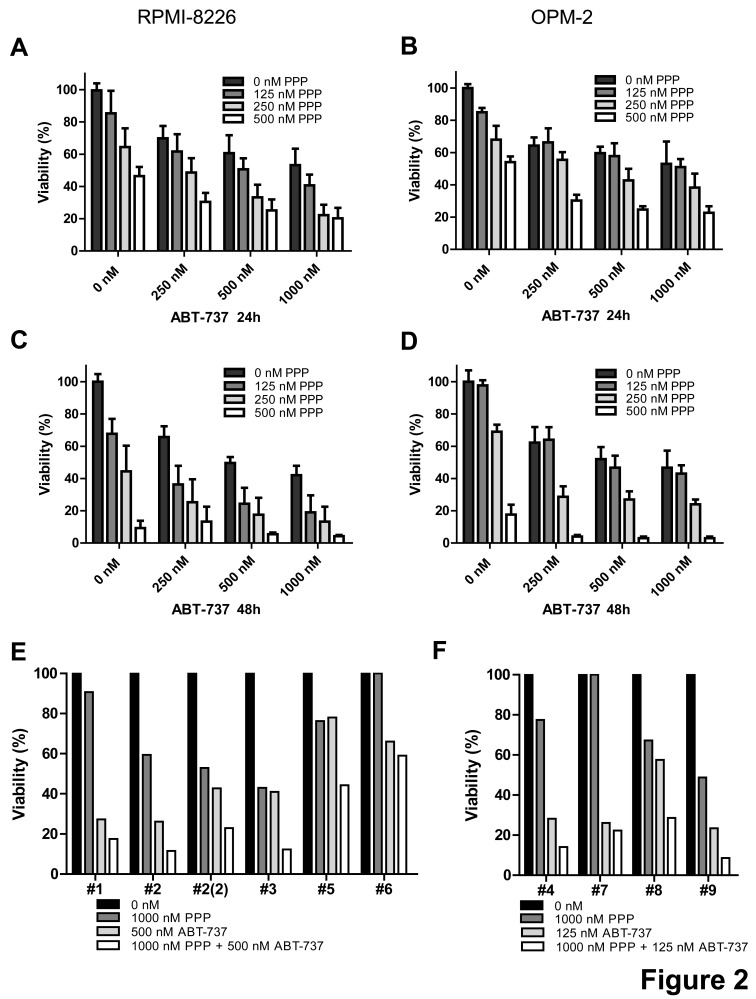
PPP synergistically enhances the anti-myeloma activity of ABT-737 A-D: PPP + ABT-737 synergistically decreased HMCL viability. RPMI-8226 (A, C) and OPM-2 (B, D) cells were cultured with 0 nM (black bars), 125 nM (dark grey), 250 nM (light grey) or 500 nM (white) PPP either alone or in combination with indicated concentrations of ABT-737 for 24h (A, B) and 48h (C, D). A-D: Effect on cell viability was evaluated by using a CellTiter-Glo Assay. Results are expressed as the relative viability compared to control cells. Bars and error bars indicate mean ± SD of at least 3 independent experiments. P-values and combination indexes after 48h are shown in Table 1. E-F: Effect of co-treatment on primary human MM cells. CD138+ cells were purified from BM aspirates from 9 MM patients. E: Primary MM cells of patient 1, 2, 3, 5 and 6 were treated with 1 μM PPP, 500 nM ABT-737 or a combination of both. F: Primary MM cells of patient 4, 7, 8 and 9 were treated with 1 μM PPP, 125 nM ABT-737 or a combination of both. The effect on cell viability was determined by the CellTiter-Glo assay after 24h. Results are expressed as the relative viability compared to untreated cells. # indicates patient number.

Next, we investigated if combined treatment with ABT-737 and the IGF-1R inhibitor PPP has synergistic anti-MM activity as compared to single agents. Based on previous reports, RPMI-8226 and OPM-2 cells were treated with a low, intermediate and high dose of either one or both agents for 24 and 48 hours [10, 19, 24, 25]. In accordance with these reports, both compounds alone reduced the number of viable cells in a dose- and time-dependent manner (Figure 2 A-D). Furthermore, co-treatment of cells significantly decreased MM cell viability as compared to single agent treated cells (Figure 2 A-D and Table 1). This was supported by combination indexes (CI) calculated to be well below 1 for most conditions, thus indicating synergistic anti-MM activity (Table 1). In parallel, studies were performed using primary MM samples from 9 MM patients (characteristics and previous treatments summarized in Supplemental Table 1). In accordance with earlier reports using primary cells, responses to ABT-737 and PPP individually varied between primary MM samples obtained from different patients [10, 19]. The results show that irrespectively of the molecular subtype or previous treatment, co-treatment consistently resulted in enhanced cell death as compared to treatment with PPP or ABT-737 alone (Figure 2 E-F). In addition, for 7 out of 9 patients, we were also able to calculate CI values and these were for most combinations below 0.5 (data not shown). Together, these data demonstrate that the IGF-1R inhibitor PPP and the BH3-mimetic ABT-737 exert synergistic anti-MM activity.

**Table 1:** Statistical analysis and combination index (CI) values

RPMI-8226	Concentration (nM)		Viability		Apoptosis	
	PPP	ABT	s	CI	S	CI
	125	250	**	0.7	***	0.63
	125	500	**	0.6	*	0.77
	125	1000	*	0.6	**	0.82
	250	250	**	0.9	***	0.74
	250	500	***	0.8	***	0.64
	250	1000	***	0.7	***	0.67
	500	250	ns	nd	ns	nd
	500	500	*	0.6	*	1.03
	500	1000	**	0.6	**	1.03
OPM-2	Concentration (nM)		Viability		Apoptosis	
	PPP	ABT	s	CI	s	CI
	125	250	ns	nd	*	0.64
	125	500	ns	nd	**	0.61
	125	1000	ns	nd	*	0.67
	250	250	***	0.7	*	0.78
	250	500	**	0.7	*	0.9
	250	1000	**	0.6	*	0.59
	500	250	*	0.7	ns	nd
	500	500	*	0.6	***	0.67
	500	1000	*	0.6	***	0.62

Significance and CI values were calculated for the different drug concentrations. Statistical significance was evaluated with a Mann-Whitney test using GraphPad Prism 5.0 software. ns = not significant, * p <0.05, ** p <0.01, *** p<0.005 when compared to both single agents. CI values were calculated by the Chou and Thalalay method using CompuSyn 1.0 software (nd = not determined, CI. 1 = synergistic).

### PPP synergistically enhances ABT-737 and ABT-199 mediated apoptosis

**Figure 3:**
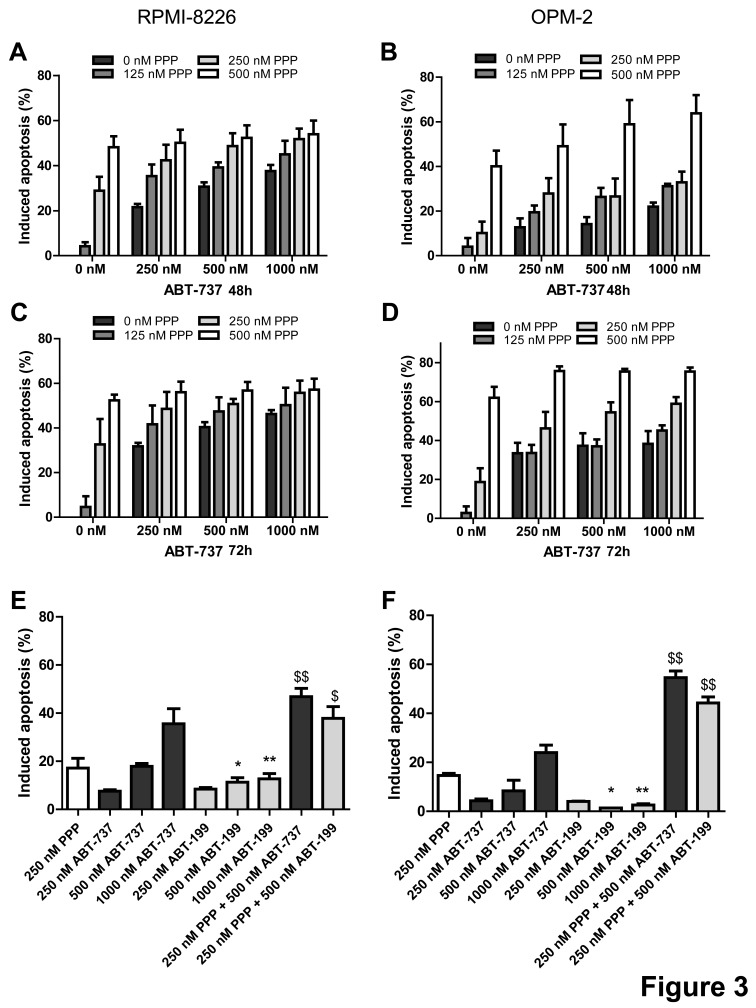
PPP potentiates ABT-737 and ABT-199 mediated apoptosis A-D: PPP increased ABT-737 mediated apoptosis. RPMI-8226 (A, C) and OPM-2 (B, D) cells were treated with 0 nM (black bars), 125 nM (dark grey), 250 nM (light grey) or 500 nM (white) PPP either alone or in combination with indicated concentrations of ABT-737 for 48h (A, B) and 72h (C, D). P-values and combination indexes after 48h are shown in Table 1. E-F: PPP increased ABT-199 mediated apoptosis. RPMI-8226 (E) and OPM-2 (F) cells were treated with 250 nM PPP either alone or in combination with indicated concentrations of ABT-737 or ABT-199 for 48h. Effect on apoptosis was determined by an AnnexinV-FITC/7′AAD staining followed by FACS analysis. Results are expressed as the percentage induced apoptosis compared to untreated cells. Columns and error bars are the mean ± SD from at least 3 individual experiments. * and ** indicate p-values of respectively ≤0.05 and ≤0.01 comparing ABT-737 with ABT-199, while $ and $$ indicate p-values of respectively ≤0.05 and ≤0.01 compared to both single agents.

**Figure 4:**
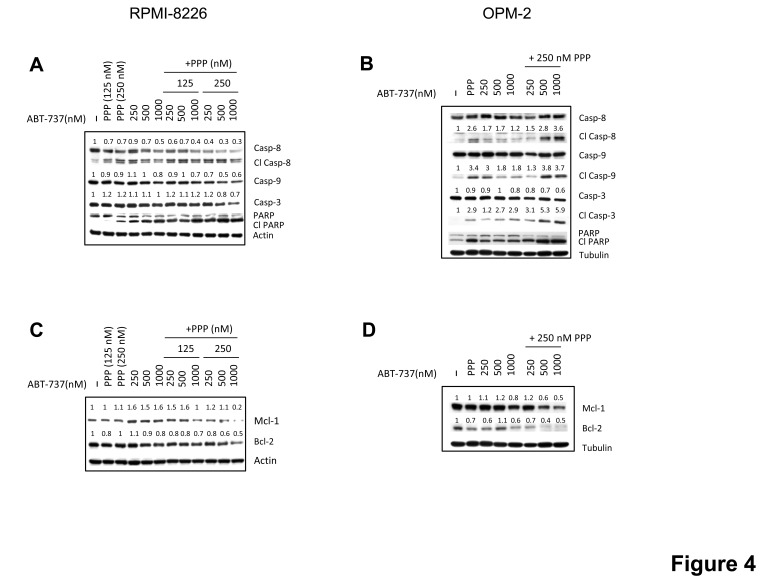
Effect of co-treatment on caspases activation and expression of Mcl-1 and Bcl-2 RPMI-8226 (A, C) and OPM-2 (B, D) cells were cultured with indicated concentrations of PPP and/or ABT-737 and the expression of Bcl-2 and Mcl-1 (24h) and cleavage of caspase-3, -8, -9 and PARP-1 (48h) was analysed by western blot. β-actin and α-tubulin were used as loading control for respectively RPMI-8226 and OPM-2 cells. One experiment representative of 3 is shown. For selected proteins, optical density was determined by ImageJ, normalized for loading control and put relative to untreated control.

We then examined the effect of the combination of ABT-737 and PPP on MM cell apoptosis. In accordance with the effect on viability, co-treatment triggered a significant increase in apoptosis as compared to either agent alone and CI values were found to be considerably lower than 1.0 (Figure 3 A-D and Table 1). Next, we also evaluated the anti-MM effect of the novel Bcl-2 specific ABT-analogue ABT-199 alone and in combination with PPP. Although we observed some induction of apoptosis, both OPM-2 and RPMI-8226 cells were less sensitive to ABT-199 than ABT-737 (Figure 3 E-F). Nevertheless, co-treatment also triggered a significant increase in apoptosis as compared to PPP and ABT-199 alone. By western blot analysis, we moreover found that co-exposure to PPP and ABT-737 clearly enhanced cleavage of caspase-8, -9, -3 and PARP-1 (Figure 4 A-B) and decreased expression of Mcl-1 and/or Bcl-2 (Figure 4 C-D) compared to either compound alone. This shows that PPP potentiates both ABT-737 and ABT-199 lethality by increasing the ABT-analogue mediated apoptosis.

### PPP overcomes the dependency of ABT-737 to Bcl-2, but fails to overcome the protective effect of Mcl-1

**Figure 5:**
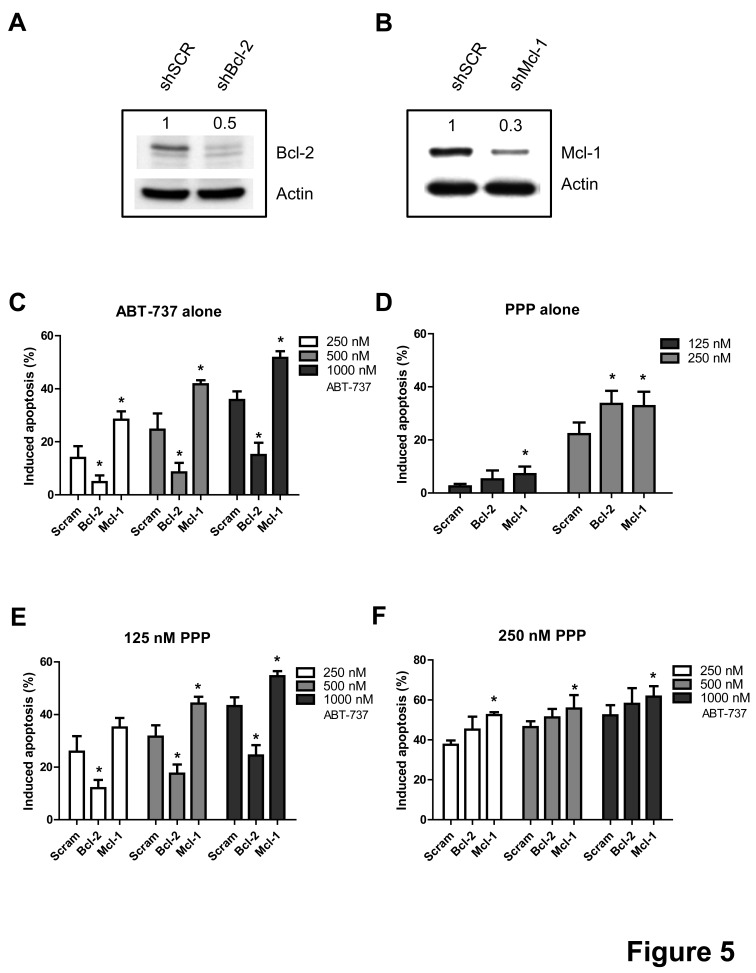
PPP overcomes the Bcl-2 dependency of ABT-737 A-B: Silencing of Bcl-2 (A) or Mcl-1 (B). RPMI-8226 cells were stably transfected with a lentiviral vector containing a shRNA cassette against either Bcl-2 or Mcl-1. Silencing of the respective proteins was confirmed by comparing expression between ShScrambled and knocked-down RPMI-8226 cells by western blot analysis. Optical density was determined by ImageJ. One experiment representative of 3 is shown. C-F: Effect of Bcl-2 or Mcl-1 silencing on the lethality induced by PPP and/or ABT-737. Cells were treated for 48h with either ABT-737 (250, 500 and 1000 nM) (C), PPP (125 and 250 nM) (D) or a combination of both (E-F) and the effect on apoptosis was evaluated by an AnnexinV-APC/7′AAD staining. Results represent the percentage induced apoptosis compared to untreated scrambled, shBcl-2 or shMcl-1 cells. Columns and error bars are the mean ± SD from 3 individual experiments. * indicates p-value of <0.05 versus ShScrambled RPMI-8226 cells.

To investigate the contribution of Mcl-1 and Bcl-2 in the synergistic anti-MM activity of PPP and ABT-737, we either silenced Mcl-1 or Bcl-2 in the RPMI-8226 cells by means of shRNA. First, the effect of shRNA on respective protein expression was confirmed by western blot analysis comparing cells transduced with a ShScrambled (control cells) and a ShBcl-2 or ShMcl-1 lentiviral vector (Figure 5 A-B). Bcl-2 silencing abrogated the response to ABT-737 (Figure 5 C). In contrast, Mcl-1 silencing resulted in a significant increase in apoptosis in response to ABT-737. In cells treated with PPP alone, both Mcl-1 and Bcl-2 silencing sensitized the cells to PPP, indicating that both anti-apoptotic proteins protect against PPP induced apoptosis (Figure 5 D). Notably, when cells were co-exposed to ABT-737 and a low concentration of PPP (125 nM, showing minimal toxicity alone), Bcl-2 silencing still significantly attenuated apoptosis although to a lesser extent as compared to control cells (Figure 5 E and Supplemental Figure 1). However, once the concentration of PPP was raised, silencing of Bcl-2 now completely failed to protect the cells against the potentiation of ABT-737 by PPP. In contrast, at both PPP concentrations, silencing of Mcl-1 further increased apoptosis compared to control cells. Together, PPP overcomes the dependency of ABT-737 to Bcl-2, but fails to overcome the protective effect of Mcl-1 in MM cells.

### *In vivo* anti-myeloma effects of ABT-737 in combination with PPP

**Figure 6:**
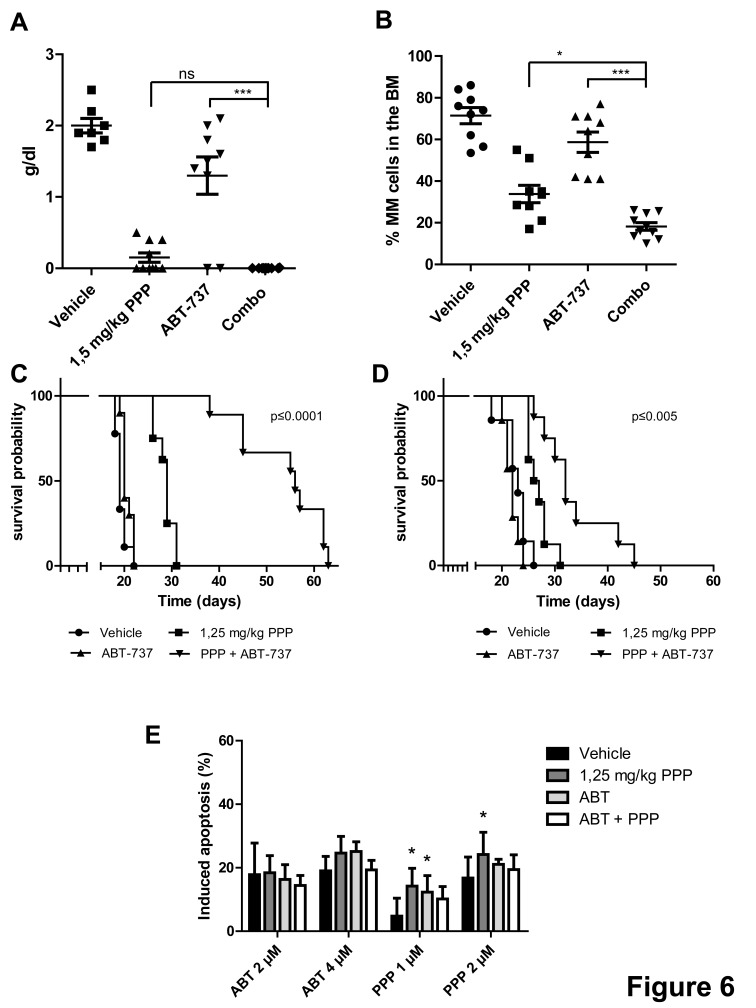
Co-treatment significantly reduces tumor burden and prolongs overall survival of 5T33MM inoculated mice A-B: *In vivo* effect of PPP and/or ABT-737 on tumor burden. 5T33MM inoculated mice were divided in 4 groups (n=10) and either treated with vehicle, 1.5 mg/kg PPP (daily in food), 75 mg/kg ABT-737 (intraperitoneal injection (i.p.) 6 days/week) or the combination of both. When the first mouse showed signs of morbidity all mice were sacrificed simultaneously and the effect on serum M-protein levels and BM plasmacytosis was determined. * p<0.05, *** p<0.001. C-D: Effect of co-treatment on the survival rates in a prophylactic (C) and a therapeutic setting (D). Mice were assigned to different treatment groups receiving either vehicle (n=10), 75 mg/kg ABT-737 (n=10), 1.25 mg/kg PPP (n=10), or a combination of both (n=10). Treatment started either 1 (prophylactic setting) or 9 day(s) (therapeutic setting) after tumor inoculation. Each mouse was sacrificed when it showed clear signs of morbidity and the effect on the survival rates was determined by Kaplan-Meier analysis. E: *In vitro* drug response of 5T33MMvv myeloma cells. Upon showing clear signs of morbidity, MM cells were isolated from mice treated either with vehicle, PPP, ABT-737 or a combination of both as described in panel D. Cells were then treated for 24h with ABT-737 (2 and 4 μM) or PPP (1 and 2 μM) and the effect on apoptosis was evaluated by an AnnexinV-FITC/7′AAD staining. Results are expressed as the percentage induced apoptosis. Columns and error bars are the mean ± SD from at least 3 individual mice. * indicate p-values of ≤0.05 compared to cells obtained from vehicle mice.

Since we observed synergistic anti-MM effects *in vitro* using HMCL, we next studied the anti-MM effects of the combination *in vivo* using the 5T33MM murine model. First, we confirmed the *in vitro* results using primary murine 5T33MM cells. IGF-1 also partially protected the 5T33MM cells against ABT-737, while PPP synergistically sensitized the cells (Supplemental Figure 2). In a first *in vivo* experiment, mice were divided in 4 subgroups and treated with: vehicle, PPP (1,5 mg/kg), ABT-737 (75 mg/kg) and the combination of PPP and ABT-737 (Figure 6 A-B). Mice treated with PPP alone showed a strong and significant reduction in BM plasmacytosis (51,4%) and serum M-protein levels (90%) compared to the vehicle group. In contrast, treatment with a suboptimal dose of ABT-737 resulted only in a modest, though significant reduction of tumor burden. However, although PPP was administrated at an optimal instead of a suboptimal dose, we still observed a significant further reduction in BM plasmacytosis in co-treated mice compared to single agent treated mice. In addition, as published earlier, PPP was found to significantly and strongly inhibit angiogenesis [18]. However, no additional effect was observed in combination treated mice (data not shown). Next, to study the effect on overall survival, a Kaplan-Meier analysis was performed either in a prophylactic (Figure 6 C) or therapeutic setting (Figure 6 D). To be able to observe any additional effect on survival compared to PPP alone, the dose of PPP was lowered to 1.25 mg/kg. In the prophylactic setting, 5T33MM mice treated with vehicle had a median survival of 19 days, whereas mice treated with PPP survived significantly longer, with a median survival of 29 days (p<0.0001). Although ABT-737 alone only prolonged the median survival of the mice with one day (p<0.01), combination of ABT-737 and PPP did significantly and strongly prolong the overall survival compared to either agent alone (median survival of 56 days, p<0.0001). Similarly, in the therapeutic setting, co-treatment of the mice was also found to significantly prolong survival (Figure 6 D). Of note, although mice were treated 6 times a week with ABT-737 and/or PPP for 5 up to 8 weeks, we detected no significant weight loss or major toxicity (data not shown). Finally, MM cells isolated from the therapeutically treated mice were also treated in vitro with different concentrations of ABT-737 or PPP to test for the possible development of acquired drug resistance (Figure 6 E). MM cells isolated from the different treatment groups were all found to respond similar to ABT-737 (and even better to PPP) compared to vehicle treated mice. Overall, these in vivo data demonstrate that co-treatment with PPP and ABT-737 decreases tumor burden and augments survival compared to treatment with either agent alone.

### Equal targeting of CD138−/CD138+ MM subpopulation

**Figure 7:**
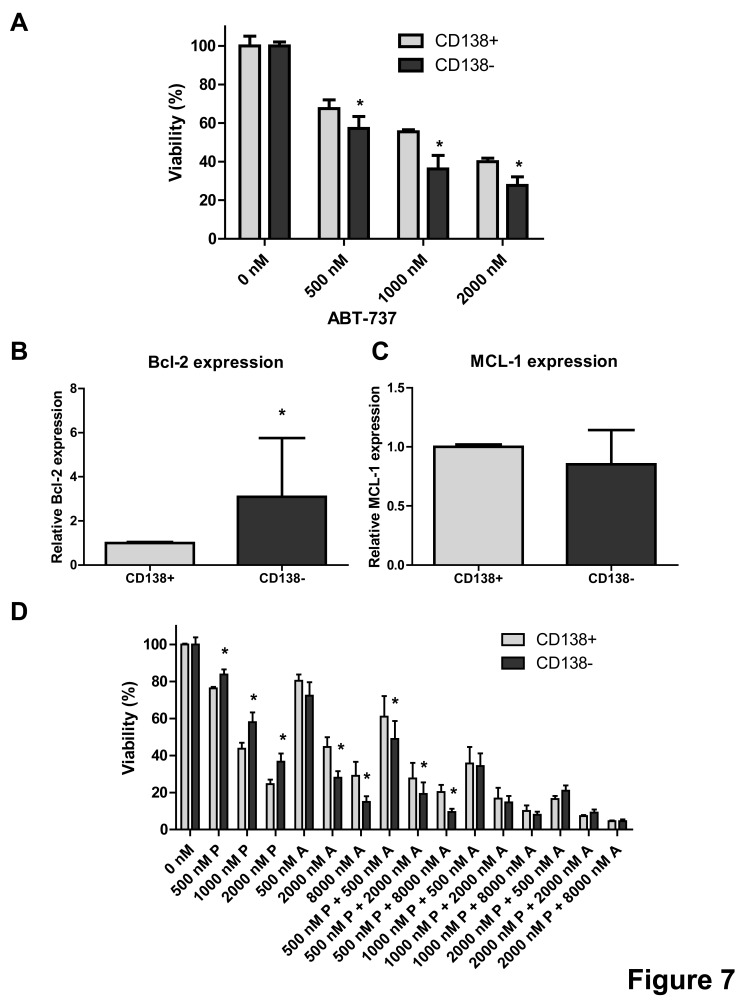
Combination of PPP and ABT-737 equally targets CD138+ and CD138− MM subpopulations A: ABT-737 sensitivity of mature CD138+ and immature CD138− 5T33MM cells. Sorted populations were incubated with different concentrations of ABT-737 (0.5, 1 and 2 μM). Cell viability was analyzed after 24h by a CellTiter-Glo assay. B-C: Relative Bcl-2 (B) and Mcl-1 (C) mRNA expression in the 2 subpopulations. mRNA expression was evaluated by quantitative realtime RT-PCR. The Bcl-2 and Mcl-1 gene expression was normalized to GAPDH. Data are presented as relative mRNA expression compared to CD138+ cells. Bars and error bars indicate mean ± SD of 4 individual mice. D: Sensitivity of the CD138+ and CD138− 5T33MM cells to ABT-737 alone and in combination with PPP. Sorted populations were incubated with PPP (0.5, 1 and 2 μM), ABT-737 (0.5, 2 and 8 μM) or a combination of both. After 24h, the viability was measured by a CellTiter-Glo assay. Results are given as the percentage viability relative to control. Bars and error bars indicate mean ± SD of at least 3 independent experiments. * indicates p value of <0.05, ** p value of <0.01 comparing CD138− cells against CD138+ cells.

Recently, it has been demonstrated that different immature CD138−negative (CD138−) MM subpopulations might exist within patients at the time of diagnosis and may mediate resistance to proteasome inhibitors [26]. In agreement to this, we showed earlier that the CD138−5T33MM subpopulation in mice is more resistant to anti-MM agents, such as the proteasome inhibitors bortezomib and MG132 [27]. Thus, we evaluated the effect of ABT-737, PPP and the combination on the CD138− and CD138+ MM subpopulations. 5T33MMvv cells were sorted in CD138− and CD138+ MM subpopulations and treated with different concentrations of ABT-737. The CD138− population was more sensitive to ABT-737 than the CD138+ population (Figure 7 A, D). In addition, the CD138− population expressed higher levels of Bcl-2 as compared to the CD138+ cells, whereas Mcl-1 levels were similar (Figure 7 B-C). In contrast to ABT-737, the CD138+ population was more sensitive to PPP than the CD138− population (Figure 7 D). In the combinations using 500 nM PPP, the CD138− population was still found to be more sensitive than the CD138+ population. However, in the combinations using higher concentrations of PPP both populations were targeted equally. Consequently, these results suggest that ABT-737 either alone or in combination with PPP could be used to target proteasome inhibitor resistant cells.

## DISCUSSION

Despite recent introduced new treatment strategies, MM remains most often incurable. Like in most cancers, elevated expression of anti-apoptotic Bcl-2 proteins such as Mcl-1, Bcl-2 and Bcl-Xl causes resistance to spontaneous or drug-induced apoptosis in MM [2, 4]. Different BH3-mimetics, including ABT-737, were extensively tested in (pre)clinical settings and their therapeutic potential was suggested in several hematological malignancies [2, 4, 5, 28]. ABT-737 and its orally bioavailable analogue ABT-263 are potent, selective small-molecule inhibitors of Bcl-2/Bcl-Xl, but not Mcl-1 [29]. Only very recently, a new orally available high-affinity Bcl-2 selective BH3 mimetic, namely ABT-199, was developed and was shown to have a better toxicity profile, while being equally as effective or even slightly superior in hematological malignancies depending mainly on Bcl-2 (such as CLL and non-Hodgkin lymphoma) [8, 9]. However, in contrast to these malignancies, MM is mainly characterized by the over-expression of Mcl-1 and thus Mcl-1 dependency [30, 31]. Mcl-1 is located on chromosome 1q21 and gains of 1q21 are unfavorable genetic prognostic factors for MM patients [32, 33]. Consequently, ABT-737 and ABT-199 are only highly effective in the CCND1 molecular subgroup presenting a Bcl-2^high^/Mcl-1^low^ profile, with ABT-199 being slightly superior to ABT-737 [10-12]. However, for all the remaining MM subgroups this superiority was less evident. In fact, an independent study even suggested that both primary MM cells and HMCL are less sensitive to ABT-199 than ABT-737, irrespective of the Bcl-2 dependency or molecular subgroup [34]. This is in line with our data showing that both RPMI-8226 and OPM-2 are less sensitive to ABT-199 than ABT-737. Consequently, since the CCND1 group only represents a small fraction of all MM cases, the ABT-analogous thus hold little promise as single agents in MM. IL-6 and IGF-1 are 2 major survival factors in MM known to regulate expression of anti-apoptotic Bcl-2 proteins [13-15, 17]. Thus, we hypothesized that IGF-1 might exert protective effects against ABT-analogous and that the IGF-1R inhibitor picropodophyllin (PPP) might potentiate the efficacy of this class of BH3 mimetics in MM. In the past, we demonstrated that PPP is a promising anti-MM agent, especially in combination with the histone deacetylase inhibitor LBH589 [18, 35].

First, we demonstrated that soluble (growth) factor(s) present in serum mediate resistance to ABT-737 and that this is partially IGF-1 mediated. These data are in line with the study of Trudel et al. who reported that growth factors and BMSC attenuate the anti-MM effect of ABT-737 [25]. In addition, yet another independent study suggested only very recently an important role for IL-6 in the BMSC mediated resistance to ABT-737 [36]. Collectively, all these data demonstrate that MM growth factors attenuate the anti-MM activity of the ABT-analogous and provide the rational for combining them with the IGF-1R inhibitor PPP. Here, we provide evidence that PPP indeed synergistically sensitized HMCL and murine 5T33MM cells to ABT-737 and ABT-199 by further decreasing cell viability and enhancing caspase-mediated apoptosis as compared to either agents alone. In support of the data, it was recently communicated that both the PI3K inhibitor LY294002 and the MEK inhibitor U0126 (targeting the two main signaling pathways activated upon IGF-1 treatment) strongly sensitized the HMCL KMS18 to ABT-737-induced apoptosis [36]. The sensitization of MM cells to ABT-analogous by PPP was furthermore confirmed using primary MM cells from 9 patients with very divergent characteristics and treatment history (see Supplemental Table 1). In conjunction to earlier reports on primary MM cells, a heterogeneous response was seen with respect to ABT-737 sensitivity [10, 25]. Consistently, however, co-treatment enhanced cell death irrespective of the molecular subtype or previous treatment of the patients. For 7 out of 9 patients, we moreover confirmed the synergistic interaction. For the remaining 2 patients, we were unable to calculate the CI values because of the limited number of cells obtained.

In hematological malignancies several compounds have already been demonstrated to have the ability to sensitize cancer cells to ABT-737-induced cell death either through Mcl-1 down-regulation or up-regulation of the endogenous Mcl-1 inhibitor Noxa [37-39]. In MM, we have demonstrated that sorafenib may improve the efficacy of ABT-737 in MM by reducing the expression of Mcl-1 [40]. In addition, arsenic trioxide synergizes with ABT-737 by inducing Noxa [41]. In agreement with previous reports, we showed that silencing of Bcl-2 strongly abrogated ABT-737 induced lethality, whereas silencing of Mcl-1 instead sensitized the cells to ABT-737 [10, 12, 24]. These data confirm that the ABT-737 (and -199) efficacy in MM depends on a high and low expression of respectively Bcl-2 and Mcl-1. In addition, we demonstrated that both Bcl-2 and Mcl-1 protect against PPP. However, co-exposure to PPP and ABT-737 overcame the Bcl-2 dependency of ABT-737, but failed to completely overcome the protective effect of Mcl-1. Based on recently communicated data that IL-6 treatment results in the phosphorylation of Bim at serine 69 and subsequent increases binding of Bim to Mcl-1, it is however still possible that PPP sensitizes MM cells to ABT-737 by post-translational modification of Mcl-1 and/or Bim thus inhibiting protein-protein binding [42]. In addition, both in MM and Bcl-2 diffuse large B-cell lymphoma ABT-737 was reported to induce autophagy and this ABT-737-induced autophagy was cytoprotective [43, 44]. Consequently, disruption of autophagy by chloroquine sensitized cells to ABT-737 and this event was accompanied with a clear increase in Bim expression in MM. Since we earlier reported that Bim expression is down-regulated by IGF-1 [13], it might be interesting to investigate in the future whether PPP is able to inhibit ABT-737 induced autophagy.

Finally, we also confirmed the enhanced anti-MM activity in vivo using the 5T33MM model. Since MM cells grow in a syngeneic BM microenvironment and the model displays biological and clinical characteristics similar to the human disease, this model is well suited for evaluating the benefits of drug combinations in vivo [21]. Here, when used at a suboptimal dose, ABT-737 alone had only minor anti-MM effects. In most xenograft MM models, both ABT-737 and -263 were also reported to fail as single agents in MM [45-47]. However, both in a prophylactic and a therapeutic setting, we demonstrated that co-treatment significantly decreased tumor burden and prolonged overall survival of the mice compared to single agent treatment. To test whether the therapeutically treated mice developed acquired drug resistance, the in vitro response of MM cells isolated from different treatment groups was tested. We found no major differences in the response of the MM cells to PPP nor ABT-737, indicating that the treatment did not alter drug sensitivity.

Recently, the presence of immature CD138− MM subpopulations within one patient was demonstrated and these were shown to mediate resistance to proteasome inhibitors [26]. Since we also demonstrated that 5T33MMvv CD138− (immature) and CD138+ (mature) subpopulations display a different in vitro sensitivity profile against a panel of drugs and both subpopulations contribute to disease propagation [27], we finally compared sensitivity of the two subpopulations to ABT-737 alone and in combination with PPP. The immature CD138− 5T33MM cells expressed higher Bcl-2 mRNA levels and were more sensitive to ABT-737 compared to the CD138+ subpopulation. In support of this, ABT-263 was also reported to selectively eradicate the quiescent ROS-low subpopulation in leukemia [48]. In contrast, PPP alone targeted the CD138+ cells more effectively. After co-treatment, both populations were targeted equally. Consequently, these data provide the rationale to implement an ABT-analogue alone or in combination with PPP in the treatment of MM after induction therapy with proteasome inhibitors. In support of this, IGF-1 was recently reported to contribute to acquired bortezomib resistance and PPP was shown to overcome this [49].

In conclusion, combination of the IGF-1R inhibitor PPP and an ABT-analogue has synergistic in vitro and in vivo anti-MM activity. Combination of an IGF-1R inhibitor (PPP) with an ABT-analogue might be a promising new approach for the design of early phase clinical trials to improve treatment of MM patients.

## MATERIAL AND METHODS

### Cell lines

The human MM cell lines OPM-2 and RPMI-8226 (obtained at ATCC, Molsheim, France) were cultured in RPMI-1640 (Lonza, Basel, Switzerland) medium supplemented with 10% FCS (Biochrom AG, Berlin, Germany), 100 U/mL penicillin/streptomycin and 2 mM L-glutamine (Lonza). The identity of the cell lines was regularly checked by short-tandem repeat analysis. Cell lines were regularly checked for infections.

### 5T33MM model

C57BL/KalwRij mice (Harlan, Horst, the Netherlands) were housed and treated following conditions approved by the ethical committee (License no. LA1230281). The 5T33MM model originated spontaneously in elderly C57BL/KalwRij mice and has ever since been propagated by intravenous transfer of the diseased marrow into young syngeneic mice [20]. For the in vivo experiments, mice were intravenously inoculated with 5×10^5^ 5T33MM cells. 5T33MMvv cells were purified from diseased mice as previously described [20] and were cultured in RPMI-1640 medium (Lonza, Basel, Switzerland) supplemented with 10% FCI (Fetal Clone I, Hyclone, South Logan, UT, USA), 1% natriumpyruvate, 1% minimum essential medium, 100U/ml penicillin/streptomycin and 2mM L-glutamine (all from Lonza).

### Primary myeloma cells

BM samples were collected for routine diagnostic or evaluation purposes after patients' informed consent and in accordance with the Declaration of Helsinki. All research was approved by the local ethical committee (B.U.N. 143201316382). BM mononuclear cells were obtained after Ficoll density gradient centrifugation (Nycomed, Lucron Bioproducts, Zurich, Switserland) and purified using MACS Cell Separation MS Columns (Miltenyi Biotec, Bergisch-Gladbach, Germany) and CD138 microbeads according to the manufacturer's protocol. Purity of the samples was evaluated by flow cytometry (FACS Canto, BD Biosciences, Franklin Lakes, USA) and analysed using FACSDiva software (BD Biosciences).

### Drugs

For *in vitro* studies, ABT-737 (Abbott Laboratories, North Chicago, USA) and picropodophyllin (PPP, Karolinska Institute, Sweden) were dissolved in dimethylsulfoxide. For *in vivo* use, ABT-737 was dissolved in solvent containing 25% polyethyleenglycol, 65% of 5% dextrose and 10% Tween-80 (Sigma-Aldrich, St.Louis, USA) and PPP was mixed in the food as previously described [18]. The concentrations of the solvents corresponding to the highest concentrations of PPP and ABT-737 were lower than 5-10%.

### Cell viability assay

Viability was measured using the CellTiter-Glo Luminescent Cell Viability assay (Promega, Fitchburg, MI, USA). This assay measures ATP as an indicator of the number of viable cells and is therefore a highly sensitive method for assaying both cell proliferation and cytotoxicity. The luminescent signal produced is proportional to the number of viable cells present. Briefly, CellTiter-Glo substrate was added and allowed to incubate at room temperature for 15 minutes after which the bioluminescent signal was quantified with the GloMax 96 plate luminometer (Promega). Experiments were performed in triplicate.

### Apoptosis assay

Apoptosis was determined by an AnnexinV/7′AAD staining followed by flow cytometric analysis (FACS Canto, BD Biosciences). Cells were washed twice with cold PBS. Next, 100 μl AnnexinV binding buffer was added together with 2 μl AnnexinV-FITC and 2 μl 7′AAD and incubated for 15 minutes in the dark at room temperature.

### Western blotting

Western blot was performed as described previously [13]. All antibodies were purchased by Cell Signaling Technology (Danvers, USA) except for Bcl-2 (sc-819) (Santa Cruz, CA, USA).

### FACS sorting of CD138^−^ and CD138^+^ MM subpopulations

5T33MMvv cells were incubated for 30 minutes at 4°C with anti-mouse CD138−PE (BD Biosciences), anti-5T33MM-idiotype [21] and CD11b-FITC antibody in buffer (PBS/2 mM EDTA/0,5 % BSA). Next, cells were washed and incubated with rat-anti-mouse IgG1-APC. Finally, cells were resuspended in buffer, 7′AAD was added and CD11b-/7′AAD-/anti-idiotype^+^ cells were sorted into CD138− and CD138+ populations using a FACSAriaI (BD Biosciences).

### Lentiviral vector production

The pLVH-CMV-eGFP-Scrambled construct was a kind gift from S. Bonné (BENE, VUB, Belgium), while the pLVH-CMV-eGFP-shBcl-2 and pLVH-CMV-eGFP-shMcl-1 plasmids were obtained from T.J. Bos (HEIM, VUB, Belgium) [13]. Lentiviral vector particles were produced in 293T cells by transient cotransfection of the transfer, envelope (pMD.G) and packaging (pCMVΔR8.9) plasmid as previously described [22]. The vector stock was collected 48 and 72 hours after transfection and concentrated by ultracentrifugation. Titer was determined by infection of 293T cells with serial dilutions of the vector stock. Seventy-two hours after infection, the number of eGFP-positive cells was determined by FACS. For transduction, 5×10^5^ RPMI-8226 cells were used. Two weeks later, cells were eGFP-sorted using the FACSAria I.

### Realtime RT-PCR

RNA was extracted with the RNeasy kit (Qiagen, Hilden, Germany) and 1 μg of total RNA was reverse transcribed using the Verso cDNA synthesis kit (Thermo Scientific, Waltham, MA, USA) according to the manufacturer's instructions. Quantitative realtime RT-PCR was performed in an ABI PRISM 7700 sequence detector (Applied Biosystems, Foster City, CA, USA). The samples were amplified in 25 μl reactions with gene specific primers for BCL-2 (forward: 5′ CTGGTTGAATGAGTCTGGGCTTTG 3′ and reverse: 5′ AGTGTTGGAGGTCTGGTGCT TAC 3′) and MCL-1 (forward: 5′ GCGTGTTATGCTCCCAGTTCC 3′ and reverse: 5′ TGCCAATCCAAGAATGCCAATCC 3′) and Maxima SYBR Green/ROX qPCR Master Mix (Thermo Scientific). Primers were purchased at Integrated DNA Technologies (Leuven, Belgium). The thermal cycling conditions included 2 minutes at 50°C and 10 minutes at 95°C, followed by 40 cycles of 95°C for 0.15 minutes and 60°C for 1 minute. The standard curve method was used to quantify gene expression of BCL-2 and MCL-1 normalized to the endogenous reference gene GAPDH (Taqman assay on demand from Applied Biosystems).

### *In vivo* experiments

To assess the effect on tumor burden, mice were assigned to different treatment groups receiving vehicle, 75 mg/kg ABT-737 (intraperitoneal injection, 6 days/week, *n*=10), 1.5 mg/kg PPP (mixed in the food, n=10) or the combination (n=10). When the first mouse showed signs of morbidity (paralysis of the hind legs), all mice were sacrificed. Serum M-protein concentration was measured by protein electrophoresis and BM plasmocytosis was determined on May-Grünwald Giesma-stained cytosmears of BM cells. For the survival study, mice received vehicle, 75 mg/kg ABT-737 (n=10; daily intraperitoneal injection), 1.25 mg/kg/day PPP (n=10), or a combination of both (n=10). Treatment started 1 day (prophylactic setting) or 9 days (therapeutic setting) after inoculation. Each mouse was sacrificed when it showed clear signs of morbidity.

### Graphical and statistical analyses

Graphical and statistical analyses were done using GraphPad Prism 5.0 software. For statistical analysis, a Mann-Whitney or Wilcoxon test was used. Kaplan-Meier analysis was conducted to create survival curves and survival probabilities were compared by a log-rank test. P values of ≤ 0.05 were considered statistically significant. Synergy was assessed by the Chou and Talalay method using CompuSyn 1.0 software [23]. A combination index (CI) > 1.0 indicates an antagonistic effect, CI = 1.0 an additive effect and CI < 1.0 a synergistic effect.

## SUPPLEMENTARY MATERIAL FIGURES AND TABLE


