# Supplementary material for: The IGF-1 receptor inhibitor picropodophyllin potentiates the anti-myeloma activity of a BH3-mimetic
Source: Oncotarget. 2014 Apr 30;5(22):11193–208. doi: 10.18632/oncotarget.1933 (PMC4294345; doi:10.18632/oncotarget.1933)
Supplement: Supplementary file 1 [file oncotarget-05-11193-s001.pdf]

The IGF-1 receptor inhibitor picropodophyllin potentiates the anti-myeloma activity of a BH3-mimetic.

Supplemental Material

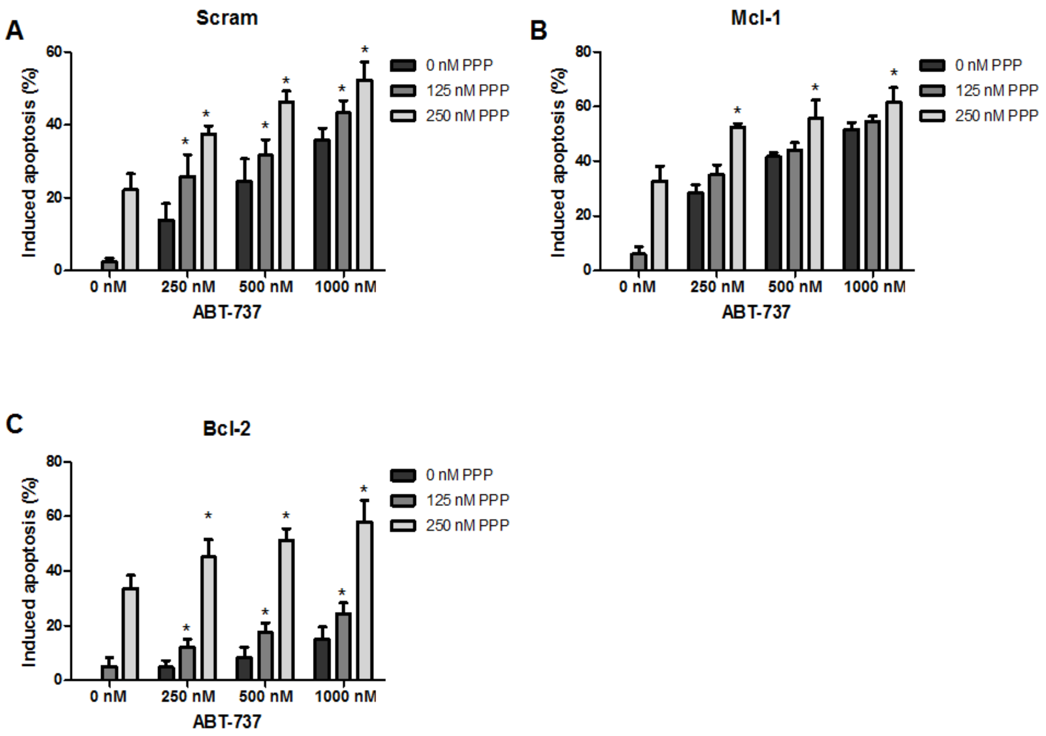

Supplemental Figure 1: Effect of the combination of ABT-737 and PPP after Bcl-2 or Mcl-1 silencing. RPMI-8226 cells were transfected with a control vector (scramble) (A) and vectors containing shRNA against Mcl-1 (B) or Bcl-2 (C). Cells were treated for 48h with ABT-737 (250, 500 and 1000 nM), PPP (125 and 250 nM) or the different combinations. The effect on apoptosis was evaluated by an AnnexinV-APC/7'AAD staining and the data is presented as the percentage induced apoptosis compared to untreated scrambled (A), shBcl-2 (C) or shMcl-1 (B) cells. Columns and error bars are the mean ± SD from 3 individual experiments. * indicates p value of <0.05 compared to both single agents.

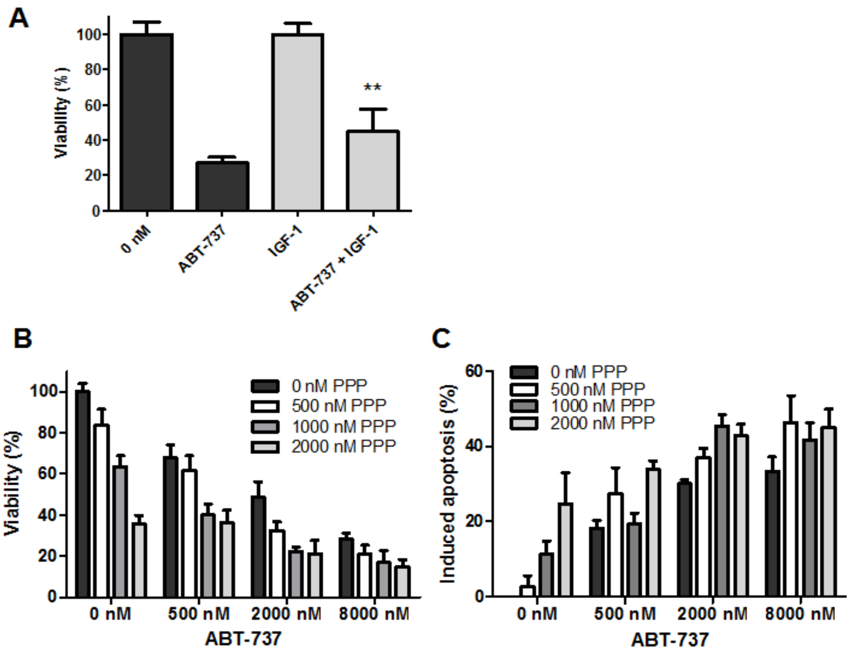

D

5T33MMvv	Concentration (nM)		Viability		Apoptosis	
	PPP	ABT	s	CI	s	CI
	500	500	ns	nd	*	0,46
	500	2000	**	0,6	*	0,37
	500	8000	*	0,7	ns	nd
	1000	500	*	0,7	ns	nd
	1000	2000	***	0,5	*	0,37
	1000	8000	*	0,6	*	0,69
	2000	500	ns	nd	ns	nd
	2000	2000	*	0,8	*	0,75
	2000	8000	*	0,7	*	0,87

Supplemental Figure 2: PPP and ABT-737 synergistically induce lethality in the murine 5T33MMvv cells. A: IGF-1 protects 5T33MMvv cells against ABT-737 induced cell death. Cells were isolated, serum starved for 1h and pre-stimulated or not with IGF-1 (200 ng/ml) for 2 hours before ABT- 737 (125 nM) was added. After 24 hours, the effect on cell viability was evaluated by using a CellTiter-Glo Assay. Results are expressed as the relative number of viable cells compared to control cells or IGF-1 condition. Bars and error bars indicate mean ± SD of 4 independent experiments. ** indicates p values of ≤0.01 comparing ABT-737 alone versus ABT-737 with IGF-1. **B-D: PPP+ABT-737 synergistically decreased MM cell viability and induced apoptosis in the 5T33MMvv cells.** Cells were cultured with 0 µM (black bars), 0.5 µM

(white), 1 μM (dark grey) or 2 μM (light grey) PPP either alone or in combination with indicated concentrations of ABT-737 for 24h. **B:** Effect on cell viability was evaluated by using a CellTiter-Glo Assay. Results are expressed as relative viability compared to control cells. Bars and error bars indicate mean ± SD of at least 3 independent experiments. **C**: Effect on apoptosis was determined by an AnnexinV-FITC/7'AAD staining. Results are expressed as the percentage induced apoptosis. Symbols and error bars are the mean ± SD from at least 3 individual experiments. **D:** Statistical analysis and combination index (CI) values. Significance and CI values were calculated for the different drug concentrations. Statistical significance was evaluated with a Mann-Whitney test using GraphPad Prism 5.0 software (ns = not significant, * $p < 0.05$, ** $p < 0.01$,*** $p < 0.005$ compared to both single agents). CI values were calculated by the Chou and Talalay method using CompuSyn 1.0 software (nd = not determined, CI<1 = synergistic).

Supplemental Table 1: Patient characteristics and previous treatment

ISS: International Staging System at diagnosis; λ: lambda, k: kappa; VAD: vincristine-doxorubicin-dexamethasone; ASCT: autologous stem cell transplantation; Vel: velcade; dex: dexamethasone; Pred: prednisolone; Rev: revlimid; Dox: doxorubicin; CTD: cyclophosphamide-thalidomide-dexamethasone; cytogenetics: molecular background at time of biopsy; ND: not determined; NA: not available.

Patient	ISS	Isotype	Previous treatment	cytogenetics
patient 1	ND	IgG λ	VAD, ASCT, ASCT, Vel-DEX-Panabinostat	NA, normal at last relapse
patient 2 (1)	3	IgG κ	Vel-DEX, ASCT	t(4;14)
patient 2 (2)	3	IgG κ	Vel-DEX, ASCT, VEL-DEX	t(4;14)
patient 3	ND	IgG κ	New	NA, loss of chr Y
patient 4	3	IgG κ	VAD, Vel-Pred, Rev-Dex	Normal
patient 5	2	Light chain κ	VAD, ASCT, Vel-Dox, Rev, Rev-Dex, VRPred, Vel-DEX, ASCT	NA, normal at last relapse
patient 6	1	IgG κ	Vel-DEX	NA, hyperdiploid karyotype at diagnosis
patient 7	3	Light chain κ	Vel-DEX, ASCT	NA, Normal at diagnosis
patient 8	2	IgG κ	New	Del13q, normal karyotype
patient 9	1	IgA λ	CTD	Del13q, karyotype ND
